# DG-AMMOS: A New tool to generate 3D conformation of small molecules using *D*istance *G*eometry and *A*utomated *M*olecular *M*echanics *O*ptimization for *in silico S*creening

**DOI:** 10.1186/1472-6769-9-6

**Published:** 2009-11-13

**Authors:** David Lagorce, Tania Pencheva, Bruno O Villoutreix, Maria A Miteva

**Affiliations:** 1 MTI, INSERM U973 - University Paris Diderot, 5 rue Marie-Andrée Lagroua, 75205 Paris Cedex 13, France; 2 Centre of Biomedical Engineering, Bulgarian Academy of Sciences, 105 Acad. G. Bonchev Str., 1113 Sofia, Bulgaria

## Abstract

**Background:**

Discovery of new bioactive molecules that could enter drug discovery programs or that could serve as chemical probes is a very complex and costly endeavor. Structure-based and ligand-based *in silico *screening approaches are nowadays extensively used to complement experimental screening approaches in order to increase the effectiveness of the process and facilitating the screening of thousands or millions of small molecules against a biomolecular target. Both *in silico *screening methods require as input a suitable chemical compound collection and most often the 3D structure of the small molecules has to be generated since compounds are usually delivered in 1D SMILES, CANSMILES or in 2D SDF formats.

**Results:**

Here, we describe the new open source program DG-AMMOS which allows the generation of the 3D conformation of small molecules using Distance Geometry and their energy minimization via Automated Molecular Mechanics Optimization. The program is validated on the Astex dataset, the ChemBridge Diversity database and on a number of small molecules with known crystal structures extracted from the Cambridge Structural Database. A comparison with the free program Balloon and the well-known commercial program Omega generating the 3D of small molecules is carried out. The results show that the new free program DG-AMMOS is a very efficient 3D structure generator engine.

**Conclusion:**

DG-AMMOS provides fast, automated and reliable access to the generation of 3D conformation of small molecules and facilitates the preparation of a compound collection prior to high-throughput virtual screening computations. The validation of DG-AMMOS on several different datasets proves that generated structures are generally of equal quality or sometimes better than structures obtained by other tested methods.

## Background

Discovery of new bioactive molecules that could enter drug discovery programs or that could serve as chemical probes to explore molecular mechanisms is very complex, time consuming and costly. In recent years, various *in silico *approaches have been reported and are now commonly used prior to or to complement experimental screening techniques with the aim of facilitating the overall process. In particular, virtual screening (VS) methods such as structure-based (SBVS) and/or ligand-based (LBVS) allow to screen thousands or millions of small molecules against a biomolecular target [1,2], and therefore, these approaches play an increasingly important role in modern drug discovery programs. SBVS makes use of docking and scoring techniques to orient and rank small molecules in the context of the protein-binding site, searching for shape and chemical complementarities [3-5]. The general concept behind ligand-based drug design relies on the molecular similarity principle that assumes that similar molecules have similar biological activity [6-8]. Both *in silico *screening methods require a suitable chemical compound collection as input. Usually, libraries should be filtered to remove compounds with non-appropriate physico-chemical properties or chemical groups causing toxicology problems (the so-called ADME-Tox filtering step) [9-11]. Further, the 3D structure of each small molecule should be generated since, for the time being, academic or commercial compound collections are most often delivered in 1D SMILES [12] (simplified molecular input line entry system), CANSMILES [13] (canonical smiles) or in 2D SDF [14] (structure data file) formats. For some of the methods, for instance rigid ligand docking or for 3D ligand-based screening experiments, a multiple conformer ensemble is also required.

It is well known that generating an accurate 3D structure for a small chemical compound is not trivial [15]. Several techniques using rule-based methods (approaches based essentially on structural data) or data-based methods [15,16] have been developed and a number of studies have been carried out in order to compare the existing approaches and to analyze the small molecule conformational preferences [17-20]. Several well established commercial packages such as Corina [21], Omega [22], Catalyst [23] or MED-3DMC [24] generate single or multiple 3D conformation of small molecules applying various approaches, including algorithms that build linker regions on the fly and combining them with pre-generated fragment libraries for the ring systems [22,25] and purely stochastic methods [26] among others [24,27-30]. In addition, several online services provide direct 2D to 3D facilities, such as OpenEye' Omega, Molsoft [31], Corina, and from academic sites such as [32,33]. Recently the web-service Frog (a mixed rule-based data-based approach) [25] has been proposed and aims at providing on-line generation of a single or ensembles of 3D conformation for drug-like compounds.

Yet, very few standalone tools generating single (or multiple) 3D conformation for a large number of compounds are freely available. The freely available Balloon program [26,34] proposes generating conformer ensembles for small molecules using a multi-objective genetic algorithm (GA) approach. We have recently developed the program Multiconf-DOCK [35] for small molecule multi-conformer generation based on a systematic search that requires as starting point the 3D structure of each input molecule in mol2 format, this package is also freely available (see [36,37]).

Here, we describe a new open source program, DG-AMMOS (Additional File 1), for the generation of a 3D conformations of small molecules using Distance Geometry and their optimization via Automated Molecular Mechanics Optimization for *in silico *Screening. DG-AMMOS makes use of the program AMMP [38,39] which is available upon GNU license. AMMP is a full-featured molecular mechanics, dynamics and modeling program. It allows manipulating both, small molecules and macromolecules with a flexible choice of potentials and a simple way to analyze individual energy terms. AMMP has been recently implemented in the well-known OpenGL molecular modeling package VEGA [40]. The generated 3D single conformer for each molecule could then be additionally subjected to different multiple conformer generator packages, such as our tool, Multiconf-DOCK [36,37]. In this study, in addition to describing the DG-AMMOS program, we validate our package on compounds from the Astex dataset [41], the ChemBridge Diversity database [42] and a number of small molecules [20] with known crystal structures extracted from the Cambridge Structural Database (CSD) [43] or the PDB [44]. The comparison of DG-AMMOS with the well-known commercial package Omega [22] and the free program, Balloon, shows convincing performances of our tool. One advantage of our program is that the source code is entirely available to users. DG-AMMOS accepts a library of chemical compounds in mol2 format with protonated molecules, this step can easily be performed using the program OpenBabel [45] or the Hgene utility of the freely available myPresto package [46].

## Implementation

DG-AMMOS drives a fully automatic procedure for the generation of 3D conformation of small molecules. The package DG-AMMOS consists of several programs developed in C and Python, and makes use of the molecular simulation package AMMP [38]. AMMP can be easily embedded in other packages and incorporates a fast multipole algorithm for the efficient calculation of long range forces thereby allowing evaluation of non-bonded terms without the use of a cutoff radius thereby increasing the speed of the computations while limiting the risk of errors or biases introduced by cutoffs [47]. The AMMP force field sp4 [47], developed on the basis of the UFF potential set [48] has been applied in DG-AMMOS.

### DG-AMMOS procedure and modules

**Figure 1:**
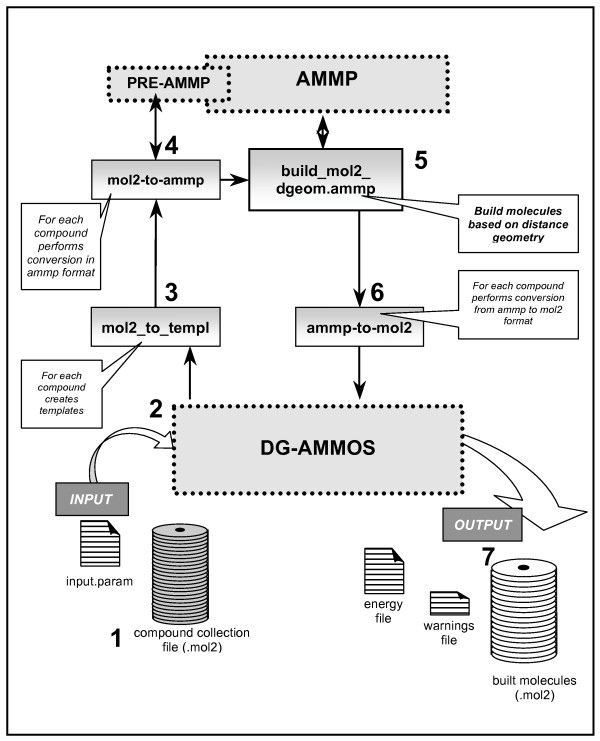
**Schematic diagram of the DG-AMMOS algorithm**. The arrows show the cycle for the automated procedure explained in details in the Implementation section.

The procedure and modules of DG-AMMOS are illustrated in Figure 1. Briefly, the architecture of the program is as follows: DG-AMMOS requires as input file a compound collection in mol2 format and returns the created 3D conformations in a mol2 format. The main python script *dg-ammos.py *ensures a full automation for a large number of small molecules. Firstly, a routine written in C, *mol2_to_templ_sp4*, creates templates required to the program PREAMMP (a part of the AMMP package), preparing the file for the specific input format *ammp *required for running AMMP. The next essential step is the generation of the 3D conformation of the small molecules by AMMP via the script *build_mol2_dgeom.ammp*. The energies of the generated structures are stored, as well as some warning messages appearing during the execution of the program. The final step is the conversion of the generated structures from AMMP format to mol2 and the saving of the results in the output files (routines written in C).

### Generation of a 3D conformation

The input structures in mol2 format may contain "zero" atomic coordinates. The input structures are treated as topology-only (2D), thus the input atomic coordinates are explicitly set to zero prior to the generation of the 3D conformation. This removes any bias introduced by the input geometry. The initial 3D conformations are constructed by DG-AMMOS using a distance geometry method which has been reviewed in details elsewhere [49,50]. The Gauss-Siedel Distance Geometry (GSDG), a hybrid Krylov solver for distance geometry, as implemented in AMMP [38] is applied in DG-AMMOS. The GSDG method takes into account bond, angle, hybrid torsion, and non-bonded point atom electrostatics and van der Waals potentials. The initial structure is corrected with molecular mechanics minimization leading to a structure with both good geometry and self-avoidance. In our implementation, we apply conjugate gradient method with the AMMP force fields sp4 [47]. The protocol employs two subsequent steps with the maximum number of iterations set to 500 and a convergence value set to 0.02 kcal.mol^-1^. Å^-1^. The resulting structures have reasonable bond lengths and valence angles provided that the optimization is allowed to iterate until convergence. To speed-up the computations, atom partial charges are assigned with the Gasteiger-Marsili method [51] using the OpenBabel package [45]. To ensure the charges' calculation for small molecules at physiological pH, we apply an in house Python script involving the OpenBabel Python module Pybel which provides the protonation of titratable groups according to a physiological pH with the Pybel option "ph = true". Users can protonate small molecules using the OpenBabel version 2.0.2 which allows to add hydrogens appropriate for pH applying the option « -p » or using the Hgene tool of the myPresto package [46]. Unrealistic structures can be generated by distance geometry, e.g., intersecting rings that are impossible to correct by using gradient based optimization methods. If unrealistic conformational energy remains after the optimization process, the structure of this particular molecule is written into a separate file.

### Program input/output

Compound libraries must be in a standard mol2 format with added hydrogens and Gasteiger charges prior to DG-AMMOS calculations. Before running DG-AMMOS, users should edit and check the parameter file, *input.param*, containing the name of the input compound collection in mol2 and the location of the DG-AMMOS package. Several output files are created, one containing the generated 3D single conformation in mol2 format (with suffix "*_Built"*) as well as a file containing the "wrong" molecules (with suffix *"_BadMolecules"*) with energy higher than 400 kcal.mol^-1 ^and a high angle and torsion energy. In addition, a table file reporting the computed non-bonded and total energy for each created structure is provided as well a warning file listing potential atom type errors.

### Data sets and program parameters

DG-AMMOS is a computer tool designed to help preparing large compound collections for subsequent virtual screening computations. In order to validate it, we decided to process a relatively large and diverse compound collection, namely the DIVERSet™ Database dated June 2009, from the ChemBridge Corporation. This database was filtered by the free FAF-Drugs2 program [11] to remove duplicates and salts, and to ensure molecules with reasonable drug-like properties (logP from -5 to 6, molecular weight from 100 to 900, and number of rotatable bonds from 0 to 20). Analysis was also performed on manually selected 114 drug-like small ligands co-crystallized with their protein targets [44] taken from the Original GOLD validation set of the Astex dataset [41,52] as well as on a set of 80 small diverse rigid compounds which we extracted from ligand-protein complexes with available X-ray structures in PDB.

In this work we run DG-AMMOS with the default parameters, these ones can be modified by the users in the file *build_mol2_dgeom.ammp*, a file that ensures the execution of the AMMP procedure for the GSDG method. In the main python script *dg-ammos.py *the energy window for a "wrong" conformation can also be selected and modified. To compare the performance of DG-AMMOS we ran also the program Balloon and generated the single 3D conformation of each compound built with DG-AMMOS. The following options were used for Balloon: "-*pStereoMutation 0*", standing for the genetic algorithm mutation probability for inverting a stereochemical center, set to 0; *"-c 1 *", standing for the number of conformers to generate, set to 1; *"-singleconf*", standing for writing only the lowest-energy conformation regardless of the population size. The MMFF94 potential energy was taken from the Balloon output files containing the generated molecule coordinates in SDF format in order to analyze the problematic structures. Omega was also run in order to validate DG-AMMOS. The algorithm implemented in Omega v.2 dissects the molecules into fragments and uses fragment templates to build a seed conformation [22]. Conformers are generated and are investigated for potential strain energy that is evaluated using the MMFF. One key parameter is the ewindow value, which defines the strain energy range within which conformers are considered as acceptable. We applied the default ewindow of 25.0 kcal.mol^-1^.

## Results and Discussion

Drug discovery is an interdisciplinary, expensive and time-consuming process and chemical biology projects share a lot of the difficulties seen in drug discovery programs. Advances in computational techniques and hardware solutions have enabled *in silico *methods, and in particular virtual screening, to speed-up modern hit identification and optimization. Both *in silico *techniques, SBVS and LBVS, often require as input chemical libraries with small molecules in 3D. Experimental sources of structural information, such as X-ray crystallography or NMR spectroscopy, remain unreachable for the millions of synthesized small chemical compounds. Thus, the need for computer-generated 3D molecular structures has clearly been recognized in drug design and chemical biology projects.

In this study we present a new open source tool for the generation of a single 3D conformation for small drug-like molecules, called DG-AMMOS, written in Python and C. The library is loaded into the engine as a mol2 file and the program outputs a file that contains the generated 3D conformation in mol2 format. The program also reports different energy values for all generated structures and a warning file is also written with possible errors appearing during the execution. In addition, "bad" molecules with very high energy are saved in a separate file, also in mol2 format. DG-AMMOS is fully automated and is user friendly. It has been tested successfully on several compound collections. The DG-AMMOS generated structures, while generally of low energy and chemically meaningful, are neither guaranteed nor intended to represent the absolute energy minimum of an input compound. This is not necessary, as DG-AMMOS-produced structures that will normally be used as the starting point for other procedures such as multiple conformer generation by our program Multiconf-DOCK [35] or docking, which will determine the lowest energy 3D conformation.

**Figure 2:**
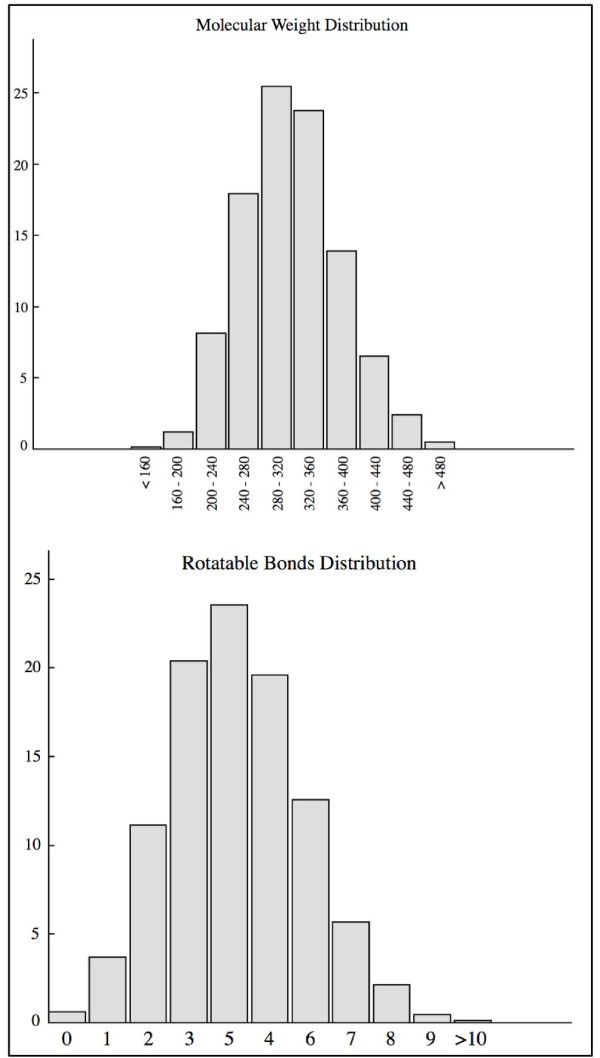
**Distribution (in %) of size and flexibility properties of the filtered DIVERSet™ Database containing 48538 drug-like molecules**. The ADME-Tox filtering and the property profiles of the filtered ChemBridge molecules were computed using the open source program FAF-Drugs2 [11] allowing prediction of ADME-Tox properties of small organic molecules.

To validate the new tool DG-AMMOS, the programs DG-AMMOS, Balloon and Omega are firstly tested on the DIVERSet™ Database initially filtered with FAF-Drug2, which resulted in 48,538 compounds. The molecular weight and rotatable bonds distributions of these compounds are shown in Figure 2. Both diagrams illustrate the diversity of the molecules in terms of size and flexibility. The resulting collection was subjected to 3D conformation generation with the three programs. The statistics for the generated structures are given in Table 1. Of the 48,538 molecules treated by DG-AMMOS and Balloon 1070 and 2915, respectively, failed because the created 3D structures showed very high energies. Visual inspection of the "wrong" molecules showed in these cases, the presence of inter-crossing cycles or non-planar aromatic cycles. Of the 48,538 initial molecules Omega generated 48,023 3D single conformers. DG-AMMOS ensured 98.7% success for the 3D generation of a large chemical library compared to 98.9% success by Omega, which confirms the robustness of our program. The filtered DIVERSet™ Database was processed by DG-AMMOS, Balloon and Omega in less than 9 h, 22 h and 3 h, respectively, on a Linux machine (Dell Precision 690, Bi-Xeon 3 Ghz processors, 2 GB DDRAM, running the CentOS 5 operating system) demonstrating that DG-AMMOS is by 3 fold more slow than Omega and by 3 fold faster than Balloon for single conformer generation.

**Table 1:** Statistics for the DG-AMMOS, Balloon and Omega run on 48538 small molecules from the ADME-Tox filtered DIVERSet™ Database.

Success	No. compounds Success (% of total database)	No. compounds Failure/(energy range in kcal/mol)	Time (s)	Time/compound (s)
DG-AMMOS	47468 (97.8%)	1074/(400 - 36230)	29739	0.61
Balloon	45623 (94.9%)	2915/(400-45944183)	79200	1.64
Omega	48023 (98.9%)	515	9481	0.20

**Figure 3:**
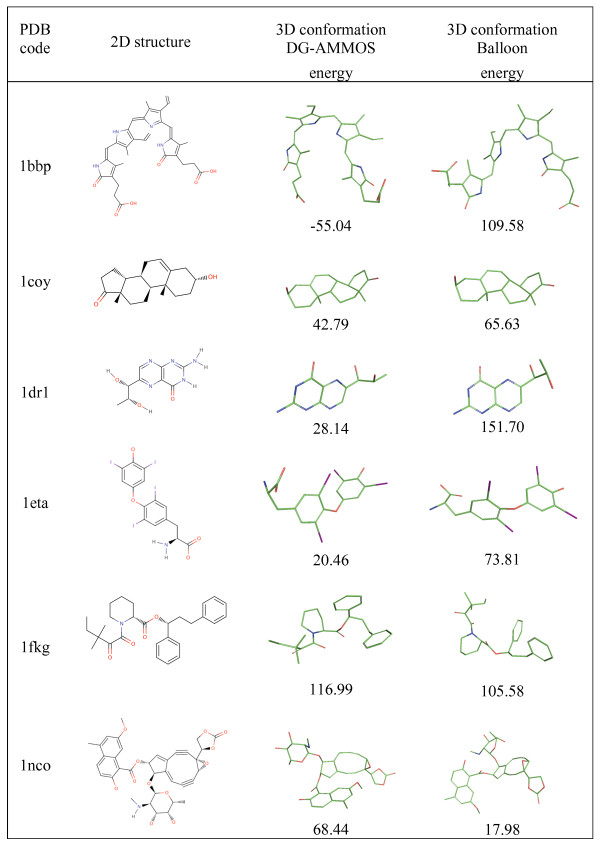
**Examples of several compounds of the selected 114 molecules from the Astex Dataset with generated 3D conformations by DG-AMMOS and Balloon**. The energies of the generated single 3D conformations (not necessarily the lowest energy ones) are shown in kcal.mol^-1^.

**Figure 4:**
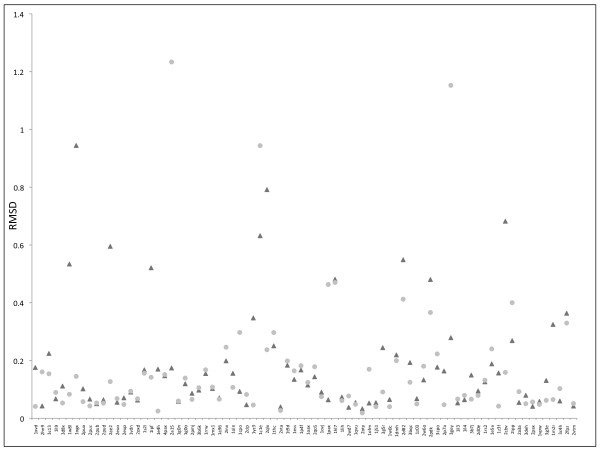
**RMSD between the single generated 3D conformers and the X-ray PDB structures for 80 rigid ligands co-crystallized with proteins**. PDB codes are given for all generated ligand structures. RMSD for conformers generated by DG-AMMOS: in triangles in black; RMSD for conformers generated by Omega: in squares in grey.

Conformer generation tools are mostly applied for modeling of protein-ligand interaction (i.e. docking) or for ligand-based lead discovery. Conformer generation algorithms are therefore typically tested on small molecules co-crystallized within a protein as found at the Protein Data Bank or against single molecule X-ray crystal structures as available from the Cambridge Structural Database. To ensure validation of the generation of bioactive small molecule 3D structures we performed computations over a set of 114 relevant compounds manually selected from the Original GOLD validation set of the Astex Dataset [41]. The Astex dataset molecules were previously chosen to assess the Balloon package. The 114 structures were successfully processed by both, the DG-AMMOS and Balloon programs. Several of these molecules are shown in Figure 3. It can be seen that both programs build correct 3D structures with "good" energy values. We performed additional testing of DG-AMMOS and Omega on a set of 80 small rigid compounds selected from ligand-protein complexes with available experimental X-ray structures in PDB [44] in order to compare the experimental structures and the generated ones in single conformer. Figure 4 presents the obtained results. Overall DG-AMMOS provides similar performance as Omega in terms of RMSD between the modeled and the experimental rigid structures. Very few compounds generated by DG-AMMOS or Omega significantly differ from the experimental ones. In most of these cases (2cbv, 1ogx, 2ae2, 1ipf, 1c1e) aliphatic cycles (considered in this study like "rigid") are generated by DG-AMMOS in different conformations compared to the X-ray structures. In the cases of 3goy and 2a15 a NHCO group is turned by 180 degree in the Omega generated single conformers, and similarly for the 2qlx conformer generated by DG-AMMOS (a turned hydroxyl group by 180 degree).

**Figure 5:**
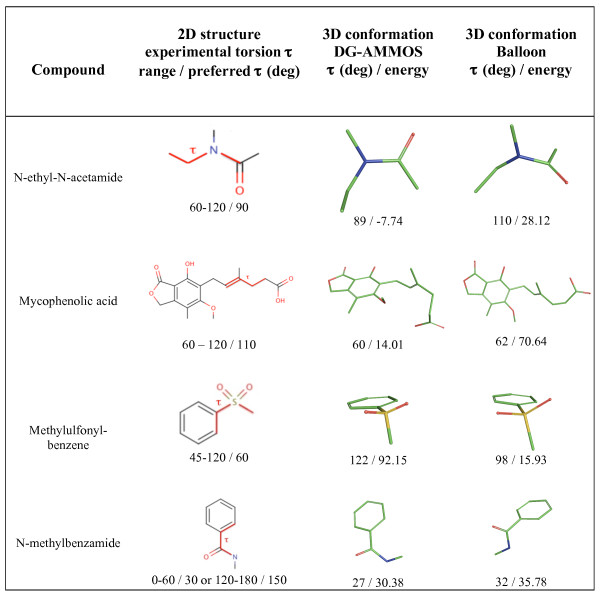
**Examples of several compounds selected from the Cambridge Structural Database representing often seen torsion angles**. These molecules contain fragments demonstrated to be part of the most represented small bioactive molecules [20]. The most preferred experimentally observed in CSD torsion angles τ and the torsion angles generated by DG-AMMOS and Balloon are given in deg. The energies are given in kcal.mol^-1^.

Along the same line of reasoning, we performed quality assessment of the generated conformers for several selected compounds extracted from the Cambridge Structural Database [20]. The results can be seen in Figure 5. For N-ethyl-N-acetamide and N-methylbenzamide both DG-AMMOS and Balloon generate torsion angles τ (in °) that correspond to the preferred ones found experimentally in the CSD. For mycophenolic acid and methylulfonyl-benzene, the predicted τ angles still remain close to the experimentally preferred torsion range. The favorable energy values obtained for the generated conformations can also be noted.

The presented results demonstrate the robustness of DG-AMMOS. All unexpected problems in terms of high energies or unknown atom types are indicated by the program. Following the widely accepted computational requirements for a conformer generator [15], DG-AMMOS is completely automated, able to easily handle large numbers of input molecules within a reasonable time and proposes high quality conformer models.

## Conclusion

DG-AMMOS provides fast, automated and reliable access to the generation of 3D structures for small drug-like molecules and greatly assists the preparation of compound collections for high-throughput virtual screening computations. Generated conformational models were investigated in terms of reliable energies, rigid structures and torsion angles. The obtained results on several different datasets prove that DG-AMMOS generated structures are generally of equal quality or sometimes better than structures obtained by other tools. DG-AMMOS requires input molecules in mol2 format, even if they do not have coordinate information, and hydrogen atoms and Gasteiger charges that can be generated by the free chemoinformatics toolkit OpenBabel. The program outputs unreasonable conformations, if any, in a separate file. The application is suitable for the generation of 3D structure for large compound collections and runs on Linux and Mac OSX platforms. The DG-AMMOS package and source code, written in Python and C, are freely available, this software is user friendly and should be a valuable addition to users working in the field of drug design.

## Availability and requirements

**• Project name: **DG-AMMOS

**• Project home page: **http://www.mti.univ-paris-diderot.fr/fr/downloads.html

**• Operating system(s): **Linux, Mac OSX

**• Programming language: **Python, C

**• Other requirements: **Python 2.5.1 to 2.6.2

**• License: **GNU GPL

## Authors' contributions

MAM designed and managed the project. DL, TP and MAM wrote the code. DL tested DG-AMMOS, Omega and Balloon. BOV and MAM optimized the program. All authors took an active part in manuscript writing, read and approved the final manuscript.

## Supplementary Material

Additional file 1 **DG-AMMOS**. This file contains the DG-AMMOS package and the user manual. Click here for file
