# Supplementary material for: DG-AMMOS: A New tool to generate 3D conformation of small molecules using Distance Geometry and Automated Molecular Mechanics Optimization for in silico Screening
Source: BMC Chem Biol. 2009 Nov 13;9:6. doi: 10.1186/1472-6769-9-6 (PMC2781789; doi:10.1186/1472-6769-9-6)
Supplement: Additional file 1 — DG-AMMOS. This file contains the DG-AMMOS package and the user manual. [file 1472-6769-9-6-S1.gz › DG-AMMOS/doc/DG-AMMOS_manual.doc]

### What is DG-AMMOS

# How to install DG-AMMOS

### How to run DG-AMMOS

### Example

### References

### What is DG-AMMOS

**DG-AMMOS** (**D**istance **G**eometry and **A**utomatic **M**olecular **M**echanics **O**ptimization for *in silico* Screening) [1] employs an automatic procedure for generation of 3D conformation of small molecules, based on distance geometry. As such, the software offers valuable solutions to assist structure-based or ligand-based *in silico* screening projects. DG-AMMOS has been developed in the laboratory of Bioinformatics-MTI, INSERM U973 – University Paris Diderot ([http://www.mti.univ-paris-diderot.fr/en/index.html](http://www.mti.univ-paris-diderot.fr/en/index.html#http://www.mti.univ-paris-diderot.fr/en/index.html)). The package DG-AMMOS is written in C and Python and is available for Linux and Mac OS X systems under the GNU General Public license. DG-AMMOS makes use of the program AMMP [2-3], available under GNU license (<http://www.cs.gsu.edu/~cscrwh/ammp/ammp.html>). Multiple conformer ensembles can be generated after DG-AMMOS by using the free tool Multiconf-DOCK ([http://www.mti.univ-paris-diderot.fr/en/downloads.html](http://www.mti.univ-paris-diderot.fr/en/downloads.html#http://www.mti.univ-paris-diderot.fr/en/downloads.html); <http://dock.compbio.ucsf.edu/Contributed_Code/multiconfdock.htm>) [4]

**Please, cite:**

# David Lagorce, Tania Pencheva, Bruno O Villoutreix, and Maria A Miteva. DG-AMMOS: A New tool to generate 3D conformation of small molecules using Distance Geometry and Automated Molecular Mechanics Optimization for in silico Screening.

You may direct questions related to this package to the corresponding author:

Maria Miteva, PhD

Senior Scientist

Bioinformatics-MTI, INSERM U973 - University Paris Diderot

5 rue Marie-Andrée Lagroua, 75205 Paris Cedex 13, France

E-mail: [maria.miteva@univ-paris-diderot.fr](mailto:maria.miteva@univ-paris-diderot.fr)

DG-AMMOS performs fast, automated and reliable access for generation of 3D conformation of small molecules and can be applied to libraries with a large number of compounds. The entire procedure of DG-AMMOS, from the input of small molecules (in mol2 format) to the final built molecules (in mol2 format) is shown in the following scheme:

**AMMP**

**mol2_to_templ**

*For each compound creates templates*

**mol2-to-ammp**

*For each compound performs conversion in ammp format*

**build_mol2_dgeom.ammp**

*Build molecules based on distance geometry*

**ammp-to-mol2**

*For each compound performs conversion from ammp to mol2 format*

**PRE-AMMP**

***OUTPUT***

input.param

compound collection

file (.mol2)

built molecules

(.mol2)

energy

file

warnings

file

**1**

**2**

**3**

**4**

**5**

**6**

**7**

***INPUT***

**DG-AMMOS**

**Figure 1**. Schematic flow of the DG-AMMOS algorithm

Back to the top

### How to install DG-AMMOS

### Step 1: Uncompress the package in a Linux shell as follows:

***> tar -zxvf DG-AMMOS.tar.gz***

The directory *~DG-AMMOS* contains the subdirectories:

- "*bin*/":	executable binary files
- "*doc*/":	user manual
- "*example*/":	an example for running DG-AMMOS
- "*install*/"
- "*progs*/":
- "*ammp*/":	the program AMMP
- "*preammp*/":	the program PREAMMP
- "*Python_scripts*/":	the Python scripts of DG-AMMOS
- "*vls_min*/":	the C programs source and input files for the
  DG-AMMOS building protocol

**Step 2: Compile the source codes**

In the *~DG-AMMOS/install* subdirectory run the compiling script "*Makefile*" by typing:

***> make***

The script will compile the source codes automatically and will generate executable files for the programs AMMP, PREAMMP, and DG-AMMOS. This script will automatically install all executable files into the directory *~DG-AMMOS/bin*/.

(*Warning*: you need Python 2.5.1 to 2.6.2)

**Step 3:**

User should edit the “.cshrc” or “.bashrc” file on a Linux operating system, or the “.profile” file on Mac OSX system, to add the path of *~DG-AMMOS/bin* in *PATH* environment variable. For example, in c-shell:

> *set path=($path ~/DG-AMMOS/bin*)

Back to the top

### How to run DG-AMMOS

In the working directory where DG-AMMOS computations will be ran, the following files should be present: *the compound collection in mol2 format* and the *input parameter file* (see for example the *input.param* file in the *~DG-AMMOS/example* directory). The user should edit this file to give the correct path and chosen collection:

The input parameter file:

path_of_*DG-AMMOS* = *path of the package DG-AMMOS^1^*

bank = *name of the compound collection in mol2 format*

**Note**:

*^1^*The total number of symbols of the path_of_*DG-AMMOS* should not exceed 80.

To run DG-AMMOS f**or** generation of 3D conformation of small molecules:

**> *dg-ammos.py*** *input_parameter_file*

DG-AMMOS Procedure:

The entire automatic procedure for generation of 3D conformation of small molecules with DG-AMMOS, from the input file (the compound collection) to the output (the final created 3D conformation of compounds) is shown in figure 1. The automatic procedure for a large number of small molecules is accomplished via a Python script. The procedure *mol2_to_templ_sp4 (available in ~DG-AMMOS/progs/vls_min)* creates template files for the small molecules, based on the initial mol2 file. Some warning could appear if unknown atom types or bonds are present. The preparation of small molecules in the specific *ammp* format is carried out with the program PREAMMP which is a part of the AMMP package.

The input file required by AMMP for the procedure of 3D conformation generation of small molecules is *build_mol2_dgeom.ammp* (available in *~DG-AMMOS/progs/vls_min*), which can be modified by the user. DG-AMMOS ensures:

1)	keeping the coordinates of the created 3D structures in mol2 format.

2)	keeping the energy of the created structures.

3)	keeping any warning that may appear during the DG-AMMOS run and “wrong” generated molecules, if any, in terms of high energy.

Back to the top

### Example

An example of generation of 3D conformation of small molecules with DG-AMMOS showing the procedure is available in the directory *~DG-AMMOS/example*. The initial molecules in mol2 format (*input_dataset.mol2*) and the input parameter file (*input.param*) (see explanations above) are provided. Users should edit the *input.param* file and then run DG-AMMOS by:

**> *dg-ammos.py input.param***

The results can be found in the directory *~DG-AMMOS/example*:

	*input_dataset_Built.mol2* contains the coordinates of built molecules (see explanations above)

	*input_dataset_BadMolecules.mol2* contains the coordinates of “wrong” molecules with high energy if any (see explanations above)

	*input_dataset_energy.txt* contains the energy of molecules (see explanations above)

	*input_dataset_total_warnings.txt* contains any warning that can appear during the DG-AMMOS run (see explanations above)

Back to the top

### References

1. **David Lagorce, Tania Pencheva, Bruno O Villoutreix, and Maria A Miteva.** DG-AMMOS: A New tool to generate 3D conformation of small molecules using Distance Geometry and Automated Molecular Mechanics Optimization for in silico Screening.
2. Harrison R., C. Reed, I. Weber. Analysis of Comparative Modeling Predictions for CASP2 Targets 1, 3, 9, and 17. *Proteins: Structure, Function, and Genetics*, 1997, Suppl. 1, 68-73
3. Weber I., R. Harrison. Molecular Mechanics Calculations on Protein–Ligand Complexes. *Perspectives in Drug Discovery and Design*, 1998, 9/10/11**,** 115-127
4. Sauton N., D. Lagorce, B.O. Villoutreix, M.A. Miteva. MS-DOCK: Accurate multiple conformation generator and rigid docking protocol for multi-step virtual ligand screening. *BMC Bioinformatics*, 2008, 9:184

Back to the top
